# RNA sequencing and integrative analysis reveal pathways and hub genes associated with TGFβ1 stimulation on prostatic stromal cells

**DOI:** 10.3389/fgene.2022.919103

**Published:** 2022-08-12

**Authors:** Peng Xiang, Zhen Du, Mingdong Wang, Dan Liu, Wei Yan, Yongxiu Hao, Yutong Liu, Di Guan, Hao Ping

**Affiliations:** ^1^ Department of Urology, Beijing Tongren Hospital, Capital Medical University, Beijing, China; ^2^ Department of Epidemiology and Biostatistics, School of Public Health, Peking University, Beijing, China

**Keywords:** benign prostatic hyperplasia, TGFβ1, prostatic stromal cells, RNA sequencing, bioinformatics analysis

## Abstract

**Objective:** Benign prostatic hyperplasia (BPH) is the most common urological disease in elderly men. The transforming growth factor beta 1 (TGFβ1) plays an important role in the proliferation and differentiation of BPH stroma. However, it is not clear yet which important pathways and key genes are the downstream of TGFβ1 acting on prostatic stromal cells.

**Methods:** GSE132714 is currently the newer, available, and best high-throughput sequencing data set for BPH disease and includes the largest number of BPH cases. We examined the TGFβ1 expression level in BPH and normal prostate (NP) by analyzing the GSE132714 data set as well as carrying out immunohistochemistry of 15 BPH and 15 NP samples. Primary prostatic stromal cells (PrSCs) were isolated from five fresh BPH tissues. RNA sequencing and bioinformatics analysis were used to reveal important pathways and hub genes associated with TGFβ1 stimulation on PrSCs.

**Results:** TGFβ1 was upregulated in BPH stroma compared to NP stroma. A total of 497 genes (244 upregulated and 253 downregulated) were differentially expressed in PrSCs with and without TGFβ1 stimulation. The Gene Ontology revealed that differentially expressed genes (DEGs) were mainly enriched in progesterone secretion, interleukin-7 receptor binding, and CSF1-CSF1R complex. The Wnt signaling pathway, PI3K−Akt signaling pathway, JAK−STAT signaling pathway, and Hippo signaling pathway were screened based on the Kyoto Encyclopedia of Genes and Genomes (KEGG) analyses. FN1, SMAD3, CXCL12, VCAM1, and ICAM1 were selected as hub genes according to the degree of connection from the protein–protein interaction (PPI) network.

**Conclusion:** This study sheds some new insights into the role of TGFβ1 in BPH stroma and provides some clues for the identification of potential downstream mechanisms and targets.

## 1 Introduction

Benign prostatic hyperplasia (BPH) is the most common urological disease in aging men, affecting approximately 50% of men at the age of 50 years (Chughtai et al., 2016). Thereafter, its prevalence increases about 10% each subsequent decade (Egan, 2016). Although the underlying etiology of BPH is still not fully understood, hormonal alterations, chronic inflammation, metabolic syndrome, and tissue remodeling related to aging have been suggested as key cofactors in the dysregulation of prostatic homeostasis (De Nunzio et al., 2016). The development of BPH is characterized by nonmalignant proliferation of the epithelial and stromal compartment in the prostate transition zone (Zhang et al., 2016). Regardless of the exact ratio of epithelial to stromal cells in the hyperplastic prostate, there is no doubt that the prostatic stromal compartment represents a significant volume of the gland.

The transforming growth factor beta (TGFβ) family plays an important role in the proliferation and differentiation of BPH stroma, as well as being a key factor for androgen-controlled prostate growth (Schauer and Rowley, 2011; De Nunzio et al., 2016). The upregulation of TGF-β1 (which is produced by prostatic stromal cells) during BPH would facilitate expansion of the stromal compartment, epithelial to mesenchymal transition, down-regulation of claudin-1, and epithelial barrier damage (Schauer and Rowley, 2011; De Nunzio et al., 2016; Wang et al., 2020; Chen et al., 2021). Moreover, TGFβ1 is one of the critical cytokines that induce fibroblasts to transform into myofibroblasts and promotes fibrosis, during which the expression of COL1A1, COL3A1, and α-SMA is increased (Sheng et al., 2018; Wang et al., 2019). The TGFβ1 expression is increased with age in the prostate (Wang et al., 2020), and the overexpression of TGF‐β1 in the murine prostate induces inflammation and fibrosis (Barron et al., 2010). Although TGFβ1 can promote proliferation and fibrosis of prostatic stromal cells, it is not very clear which important pathways and key genes are the possible downstream of TGFβ1.

RNA sequencing (RNA-seq) is a promising and widely used technology that can be used to analyze the complete characterization of RNA transcripts, including transcription start site mapping and gene fusion detection (Wang et al., 2018). In this study, we used the RNA-seq method to study primary prostatic stromal cells with or without TGFβ1 treatment, in order to reveal important pathways and hub genes related to the downstream of TGFβ1. Therefore, this study will improve our understanding of the mechanism of TGFβ1 on PrSCs, which may gain more insights into the potential therapeutic targets during the progression of BPH.

## 2 Materials and methods

### 2.1 Patient specimens and ethics statement

A total of fifteen BPH samples were derived from patients undergoing the transurethral resection of prostate (TURP). Also, fifteen normal prostate (NP) samples were acquired from patients (aged ≤50 years) undergoing cystoprostatectomy for infiltrating bladder cancer without prostate infiltration. We excluded patients with prostate cancer and prostatitis, as well as patients receiving alpha-adrenergic receptor antagonists or 5α-reductase inhibitors. All procedures performed in the research involving human participants were conducted in accordance with the principles of the Declaration of Helsinki. The study was approved by the Ethics Committee at Beijing Tongren Hospital.

### 2.2 Immunohistochemistry

The prostate tissues were fixed in 4% formalin buffer at 4°C overnight, then dehydrated in ascending ethanol series, embedded in paraffin, and cut into 5-μm sections. After conventional deparaffinization, hydration, and antigen retrieval, the endogenous peroxidase was inactivated by 3% hydrogen peroxide. The primary antibodies of rabbit anti-TGFβ1 (1: 500, Abcam) were used for incubation at 4°C overnight. The primary antibody was recognized by the biotinylated secondary antibody at room temperature for 30 min and visualized by the VECTASTAIN ABC peroxidase system and peroxidase substrate DAB kit. The TGFβ1 expression level was blindly determined via the pathological review based on the staining score (0–9) that is defined by multiplying the staining intensity score (0–3) with the staining extent score (0–3) in prostate tissues.

### 2.3 Isolation and culture of primary prostatic stromal cells

A total of five human primary prostatic stromal cells (PrSCs) were obtained from five different BPH tissues. Briefly, fresh prostatic tissues were dissected into small fragments, and primary prostatic stromal cells were isolated and cultured as described previously (Sheng et al., 2018; Chen et al., 2021). The stromal cells were cultured with RPMI 1640 (Gibco, Rockville, MD, United States) supplemented with 10% fetal bovine serum (FBS) (Gibco, Grand Island, NY, United States) and 1% penicillin–streptomycin solution (Gibco) at 37°C under 5% CO2 and humidified atmosphere. The stromal cells were used at passages 3–5. According to the previous literature, the commonly used dose of TGFβ1 in the study of benign prostatic hyperplasia ranges from 1 ng/ml to 10 ng/ml (Sheng et al., 2018; Wang et al., 2020; Wang Z. et al., 2021). Therefore, cells in our study were treated with 10 ng/ml TGFβ1 (R&D Systems, MN, United States) for 72 h.

### 2.4 RNA sequencing

RNA sequencing was performed on PrSCs with or without TGFβ1 treatment. Profiling of transcriptome analysis was performed with 2 μg high-quality total RNA per sample by RNA-seq at Annoroad Gene Technology Corporation (Beijing, China) according to the procedures described previously (Hu et al., 2018; Li et al., 2020). Briefly, total RNA was isolated using TRIzol reagent (Thermo Fisher Scientific). RNA samples were rRNA depleted, and RNA libraries were constructed using the TruSeq RNA Library Prep Kit v2 (Illumina) and sequenced as 150 bp paired-end reads using the Illumina HiSeq 2000 (Beijing Annoroad Co. Ltd.). We filtered RNA-seq next-generation sequencing (NGS) reads to obtain clean reads for further evaluation and analysis, including quality inspection of reads according to the Phred score, in comparison to the human genome reference assembly (hg19) using HiSAT2 and merger of transcripts in StringTie. We used fragments per kilobase of transcript per million mapped reads (FPKM) to assess mRNA expression. Finally, the heatmap was generated using R software with differentially expressed genes (|logFC|>1, q-value<0.05). Gene-enrichment and Gene Ontology-based functional annotation were performed with DAVID Bioinformatics Resources 6.8. A hypergeometric distribution test was carried out to identify GO (Gene Ontology) functions and KEGG (Kyoto Encyclopedia of Genes and Genomes) pathways in which DEGs were significantly enriched (q-value <0.05) compared with total background expressed genes. Next, we performed the analyses of protein–protein interaction (PPI) networks using STRING (Search Tool for the Retrieval of Interacting Genes) and Cytoscape to take aim at potential targets.

### 2.5 Statistical analysis

Statistical analyses were performed using GraphPad Prism software, version 7. Significant differences in statistical analyses were calculated using a two-tailed Student’s t-test for two groups. *p*-values <0.05 were considered to be statistically significant.

## 3 Results

### 3.1 TGFβ1 was upregulated in BPH stroma compared to NP stroma

**FIGURE 1:**
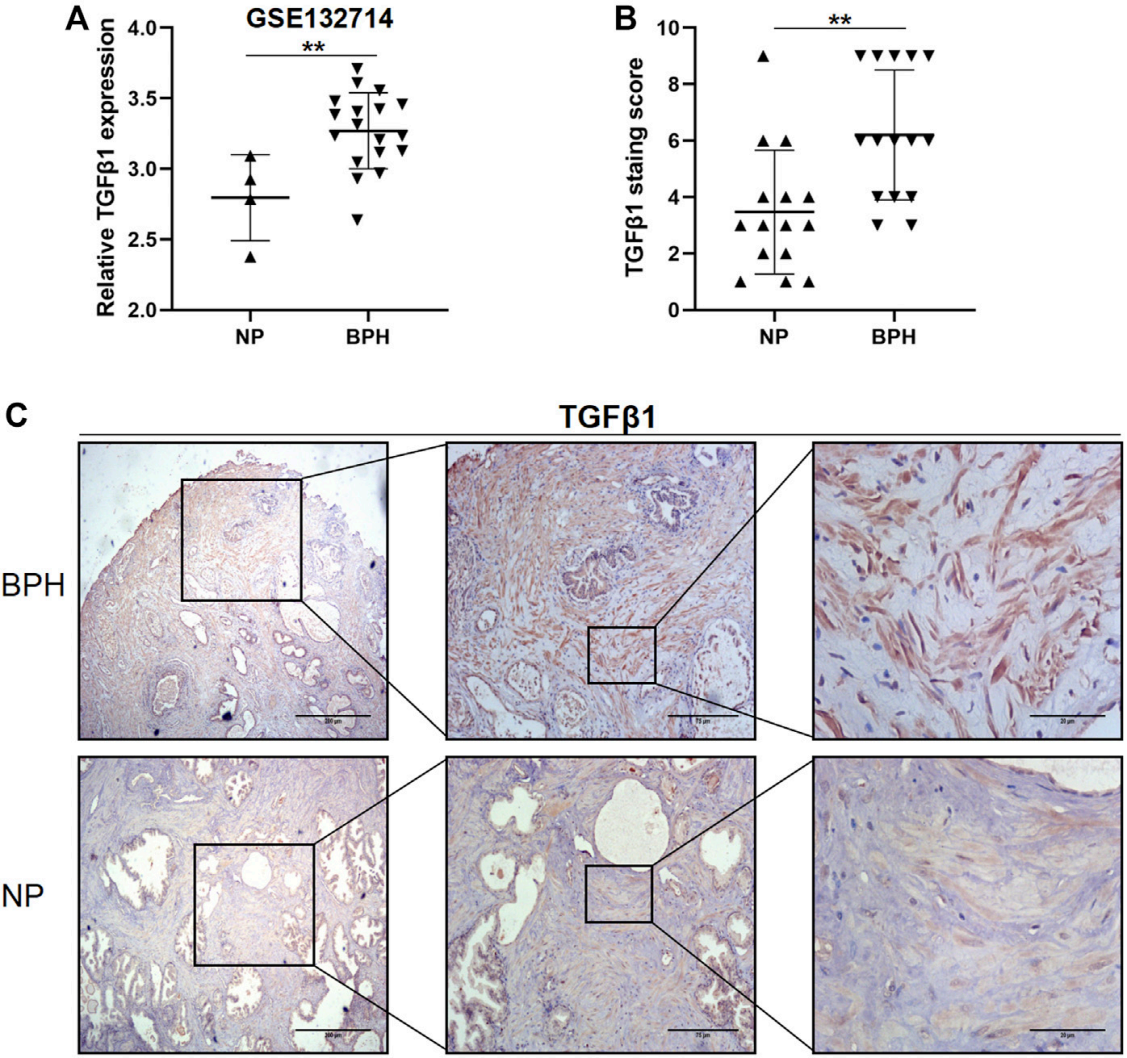
TGFβ1 is strongly upregulated in benign prostatic hyperplasia (BPH) compared with normal prostate (NP). **(A)** Expression levels of TGFβ1 mRNA between BPH and NP in the GSE132714 data set. **p < 0.01. **(B)** Expression levels of TGFβ1 protein in BPH and NP tissues. Protein expression of TGFβ1 was assayed by immunohistochemical staining in prostatic tissues. **p < 0.01. **(C)** Representative TGFβ1 immunohistochemical staining images in BPH and NP tissues at different magnification levels.

GSE132714 is currently the newer, available, and best high-throughput sequencing data set for BPH disease and includes the largest number of BPH cases. To determine the role of TGFβ1 in BPH, we first examined the relative mRNA expression level of TGFβ1 in BPH and normal prostate using this GSE132714 data set. Among 18 BPH and 4 NP tissues being analyzed, the TGFβ1 mRNA expression level was higher in BPH (*p* = 0.0054, Figure 1A). In order to determine whether the TGFβ1 protein level showed the same increasing trend in BPH, we used immunohistochemistry staining to compare the TGFβ1 protein expression in 15 BPH samples and 15 NP samples. As shown in Figures 1B,C, TGFβ1 was primarily expressed in the prostatic stroma, and the TGFβ1 protein expression was higher in BPH than the normal prostate (*p* = 0.0025).

### 3.2 Differential gene expression of TGFβ1 treatment on PrSCs

**FIGURE 2:**
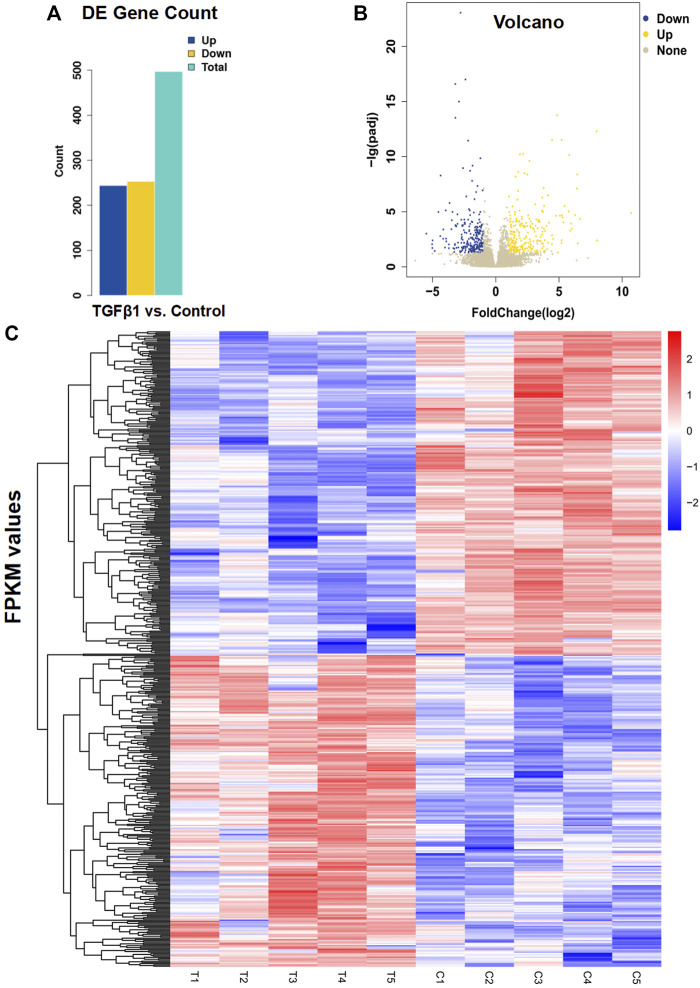
Analysis of differential gene expression in PrSCs stimulated with and without TGFβ1. **(A)** Results of differentially expressed genes (DEGs) were counted according to the screening criteria of |log2 Fold change|≥1 and q < 0.05. Of the 497 differential genes detected in PrSCs with TGFβ1 stimulation, 244 genes were upregulated and 253 genes were downregulated. T_C means TGFβ1 treatment versus control. **(B)** Volcano diagram showed significantly DEGs in PrSCs stimulated with and without TGFβ1. Yellow spots represented upregulated genes, and blue spots represented downregulated genes. Gray spots indicated genes that were not differentially expressed. **(C)** Heatmap plot of all 497 DEGs in five PrSCs with and without TGFβ1 treatment. The legend color bar on the right side indicated the relation between FPKM-scaled expression values and colors, and the colors were balanced to ensure that the white color represented a zero value. C1, C2, C3, C4, and C5 in the heatmap mean PrSCs without TGFβ1 treatment (control). T1, T2, T3, T4, and T5 in the heatmap mean PrSCs with TGFβ1 treatment.

To examine the effect of TGFβ1 on prostatic stromal cells, we first isolated primary prostatic stromal cells from five BPH samples, and Supplementary Figure S1 showed the microscopic morphology of PrSCs. Then, we performed RNA sequencing on PrSCs treated with and without TGFβ1. The results indicated that a total of 497 genes (244 upregulated and 253 downregulated) were differentially expressed between TGFβ1 treatment and control (Figure 2A). In the meantime, the volcano diagram results showed significantly DEGs between TGFβ1 treatment and control (Figure 2B). The heatmap plot of 497 DEGs is shown in Figure 2C; the top 10 significantly upregulated DEGs included COL10A1, COMP, IL11, NOX4, UCN2, SLC19A2, CALB2, TNFSF15, COL7A1, and BHLHE40, as well as the top 10 significantly downregulated DEGs included CSF1, VAMP5, SECTM1, APOL1, APOL3, GBP2, CD47, GMPR, UBA7, and FZD4. Moreover, we verified the top 10 significantly upregulated and downregulated DEGs in two primary prostate stromal cells (PrSCs) using quantitative PCR, and the results were basically consistent with the corresponding RNA-sequencing results (Supplementary Figure S2). In addition, all data of DEGs are shown in Supplementary Table S1.

### 3.3 GO classification and enrichment analysis of DEGs

**FIGURE 3:**
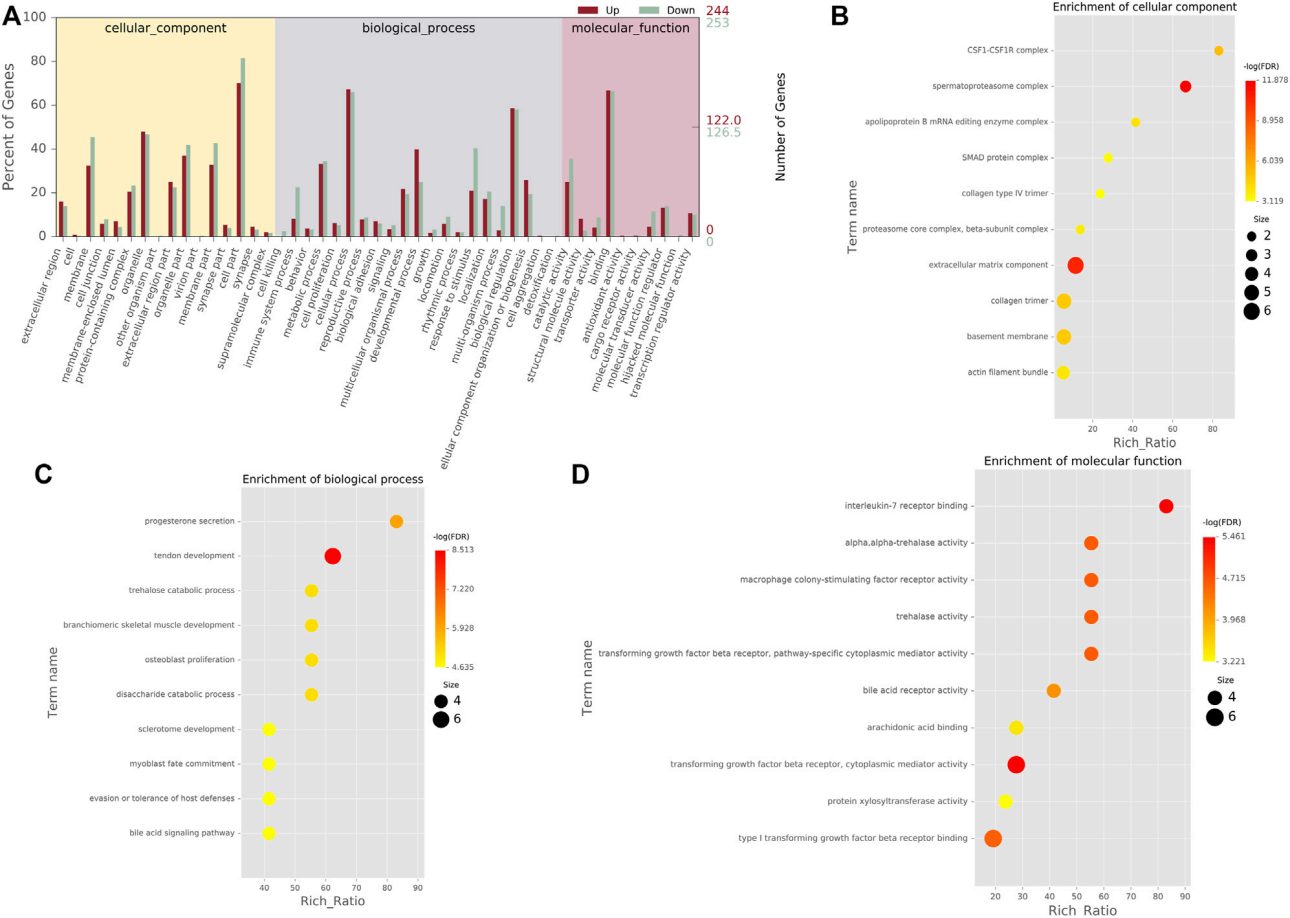
GO classification and enrichment analyses of DEGs **(A)**. GO classification of DEGs. The *x*-axis indicated the subcategories, the left *y*-axis represented the percentage of a specific category of DEGs, and the right *y*-axis indicated the number of DEGs **(B)**. Top 10 cellular component (CC) terms in the enrichment analysis **(C)**. Top 10 biological process (BP) terms in the enrichment analysis. **(D)** Top 10 molecular function (MF) terms in the enrichment analysis.

In order to determine the function of DEGs, all DEGs were mapped to terms in the GO database. This list of 497 DEGs was divided into three main categories of GO classification (e.g., biological process, cellular component, and molecular function). For biological processes, most of those were classified into cellular process, biological regulation, and metabolic process. For the molecular function category, binding, catalytic activity, and molecular function regulator were the top abundant subcategories. Under the cellular component category, a large number of upregulated, as well as downregulated DEGs were categorized as cell part, organelle, and organelle part (Figure 3A). Moreover, the cell component indicated enrichment predominantly at the CSF1-CSF1R complex, spermatoproteasome complex, apolipoprotein B mRNA editing enzyme complex, SMAD protein complex, and collagen type IV trimer (Figure 3B). DEGs were mainly enriched in biological processes of progesterone secretion, tendon development, trehalose catabolic process, branchiomeric skeletal muscle development, and osteoblast proliferation (Figure 3C). As for molecular function, these genes showed enrichment in interleukin-7 receptor binding, alpha,alpha-trehalase activity, macrophage colony-stimulating factor receptor activity, trehalase activity and transforming growth factor beta receptor, and pathway-specific cytoplasmic mediator activity (Figure 3D).

### 3.4 KEGG pathway analysis of DEGs

We performed KEGG pathway analysis of DEGs between control and TGFβ1 treatment. The results indicated that Wnt signaling pathway (*p* = 0.0039), TNF signaling pathway (*p* = 0.0002), Th17 cell differentiation (*p* = 0.0006), signaling pathways regulating pluripotency of stem cells (*p* < 0.0001), PI3K−Akt signaling pathway (*p* = 0.0017), osteoclast differentiation (*p* < 0.0001), JAK−STAT signaling pathway (*p* = 0.0003), Hippo signaling pathway (*p* = 0.0012), glycerophospholipid metabolism (*p* = 0.0001), and cytokine−cytokine receptor interaction (*p* < 0.0001) may be involved in the regulation of TGFβ1 on primary prostatic stromal cells. The KEGG results of the enrichment of 29 pathways are shown in Supplementary Figure S3. In addition, the details related to KEGG pathways are also shown in Supplementary Table S2.

### 3.5 PPI network construction and hub gene selection

Proteins related to DEGs were selected on the basis of STRING database, and the pairs whose combined score >0.7 were extracted for visualization by Cytoscape (Supplementary Figure S4). Each node displays different depth colors according to its degree score. From the inside to the outside, the degree decreases and the color changes from dark to light. Furthermore, hub genes were selected with connection degree ≥10. In this network, the top 13 genes with the highest degree scores were selected as hub genes, including FN1, SMAD3, CXCL12, VCAM1, ICAM1, PSMB8, SOCS3, CCL2, IRF1, TNFRSF1B, SOCS1, PPARG, and LPAR3. The details of hub genes are shown in Supplementary Table S3.

## 4 Discussion

In the current study, we first used the GSE132714 data set and immunostaining method to determine that TGFβ1 is highly expressed in the BPH stroma compared with the NP stroma. Then, we used RNA-seq and bioinformatics analysis to reveal important pathways and hub genes associated with TGFβ1 stimulation on primary prostatic stromal cells. This study provided evidence that the inflammatory cytokine TGF-β1 can cause a series of significant pathways and gene changes in prostatic stromal cells.

The TGFβ1 pathway is activated in BPH and contributes to increased stromal proliferation and fibrosis. However, it is not very clear about the potential significant pathways and hub genes related to TGFβ1 stimulation on PrSCs. In this study, a total of 497 DEGs were identified in PrSCs with and without TGFβ1 treatment. Then, GO and pathway enrichment analyses of DEGs were performed. Moreover, the Wnt signaling pathway, PI3K−Akt signaling pathway, JAK−STAT signaling pathway, and Hippo signaling pathway were screened based on the KEGG analysis. Additionally, we constructed the PPI network and selected FN1, SMAD3, CXCL12, VCAM1, and ICAM1 as hub genes according to the degree of connection.

All of the aforementioned hub genes play a vital role in cell cycle, proliferation, and fibrosis, which may contribute to the pathogenesis of BPH. Fibronectin (FN1) is an essential extracellular matrix glycoprotein involved in both physiological and pathological processes. Fibronectin could stimulate the proliferation of growth-arrested polarized mammary epithelial cells, induce an EMT response, disturb the hollow acinar structure, and promote tumor-like behavior (Park and Schwarzbauer, 2014; Konac et al., 2017). At the same time, FN1 is likely to play a pivotal role in fibrosis (Cardoso et al., 2018; Chen et al., 2021). It was reported that the phosphorylation of SMAD3 can promote the differentiation of fibroblasts into myofibroblasts, fibrosis, and EMT during the progression of BPH (Sheng et al., 2018; Tang et al., 2019; Chen et al., 2021). CXCL12 overexpression and secretion by aging fibroblasts could enhance human prostate epithelial proliferation *in vitro* (Begley et al., 2005). Moreover, CXCL12/CXCR4 axis activation induces prostate myofibroblast phenoconversion through non-canonical EGFR/MEK/ERK signaling (Rodriguez-Nieves et al., 2016). High vascular cell adhesion molecule (VCAM-1) expression is significantly associated with clinical stage and distant metastasis in prostate cancer (Duzagac et al., 2015; Chang et al., 2018). The JAK/STAT pathway interacts with intercellular cell adhesion molecules (ICAM-1) and VCAM-1 to promote tumor progression (Duzagac et al., 2015). However, the role of ICAM-1 and VCAM-1 in BPH has not been fully elucidated.

Wnt signaling regulates cell proliferation and cell differentiation as well as migration and polarity during development (Brunt et al., 2021). Wnt/β-catenin and AR signaling contribute to the proliferative growth of many cell types and benefit from the cross-talk within the prostate (Kypta and Waxman, 2012; Koirala et al., 2020). The status of the Wnt/β-catenin pathway in the prostate stroma may serve as a marker at various stages of BPH pathogenesis (Koirala et al., 2020). The phosphatidylinositol 3-kinase/protein kinase B (PI3K/AKT) signaling pathway promotes cell proliferation and fibrosis, as well as plays an important role in promoting the occurrence of BPH (Sheng et al., 2018; Wang S. S. et al., 2021). In addition, aerobic exercise may alleviate BPH in obese mice through regulation of the AR/androgen/PI3K/AKT signaling pathway (Wang S. S. et al., 2021). M2 macrophage-derived IL4 induced the myofibroblast phenotype through the JAK/STAT6 and PI3K/AKT signaling pathways in the early-progressed BPH prostate fibroblasts (Sheng et al., 2018). It has been demonstrated that febuxostat could ameliorate testosterone-induced BPH rats via suppressing the XO/JAK/STAT axis (Abo-Youssef et al., 2020). Furthermore, STAT-3 signaling is negatively regulated by labda-8 (17),12,14-trien19-oic acid to prevent proliferation of BPH stromal cells (Verma et al., 2014). One of the important signaling pathways that control cell growth/proliferation, cellular homeostasis, and organ development is the Hippo pathway (Park et al., 2018). In advanced prostate cancer, IKBKE activity enhances AR levels via modulation of the Hippo pathway (Bainbridge et al., 2020). Alginate oligosaccharide could attenuate α2,6-sialylation modification to inhibit prostate cancer cell growth via the Hippo/YAP pathway (Han et al., 2019). However, there is currently no research on the role of Hippo signaling pathway in BPH. Finally, our research indicated that these hub genes and differentially significant pathways may be the key for studying downstream mechanisms of TGFβ1 in PrSCs.

In conclusion, our study demonstrated a series of differentially expressed genes and pathways by bioinformatics analysis, which may contribute to the finding of molecular downstream mechanisms of TGFβ1 in the BPH stroma. Hub genes such as FN1, SMAD3, CXCL12, VCAM1, and ICAM1 may serve as the central downstream genes of TGFβ1 in BPH stromal cells. The Wnt signaling pathway, PI3K−Akt signaling pathway, JAK−STAT signaling pathway, and Hippo signaling pathway may be the key downstream pathways for TGFβ1 to exert its effect on the BPH stroma. Further molecular experiments are required to confirm the findings of this study.

## Data Availability

The data presented in the study are deposited in the GEO repository, accession number GSE205378. We have released our data. The link is as follows: https://www.ncbi.nlm.nih.gov/geo/query/acc.cgi?acc=GSE205378.

## Supplementary material

Supplementary Figure S1 Microscopic morphology of primary prostatic stromal cells.

Supplementary Figure S2 Verification of RNA-seq results by qPCR. A and B: qPCR comparison of 10 significantly upregulated DEGs in PrSCs with TGFβ1 and without TGFβ1 (Control). C and D: qPCR comparison of 10 significantly downregulated DEGs in PrSCs with TGFβ1 and without TGFβ1 (Control).

Supplementary Figure S3 Scatter plot for KEGG enrichment results. The significant 29 enrichment pathways are shown in the senior bubble chart. The Rich Ratio is the ratio of DEG numbers annotated in this pathway term to all gene numbers annotated in this pathway term. A Q-value is the corrected p-value.

Supplementary Figure S4 Protein–protein interaction network for DEGs. Each node displays different depth colors according to its degree score. From the inside to the outside, the degree decreases and the color changes from dark to light.

Supplementary Table s1 All details of DEGs in PrSCs with and without TGFβ1 treatment.

Supplementary Table S2 Details related to KEGG pathways.

Supplementary Table S3 Top 13 genes in the protein–protein interaction network (node degree ≥ 10).
