# Supplementary figures and images for: RNA sequencing and integrative analysis reveal pathways and hub genes associated with TGFβ1 stimulation on prostatic stromal cells
Source: Front Genet. 2022 Aug 12;13:919103. doi: 10.3389/fgene.2022.919103 (PMC9412917; doi:10.3389/fgene.2022.919103)

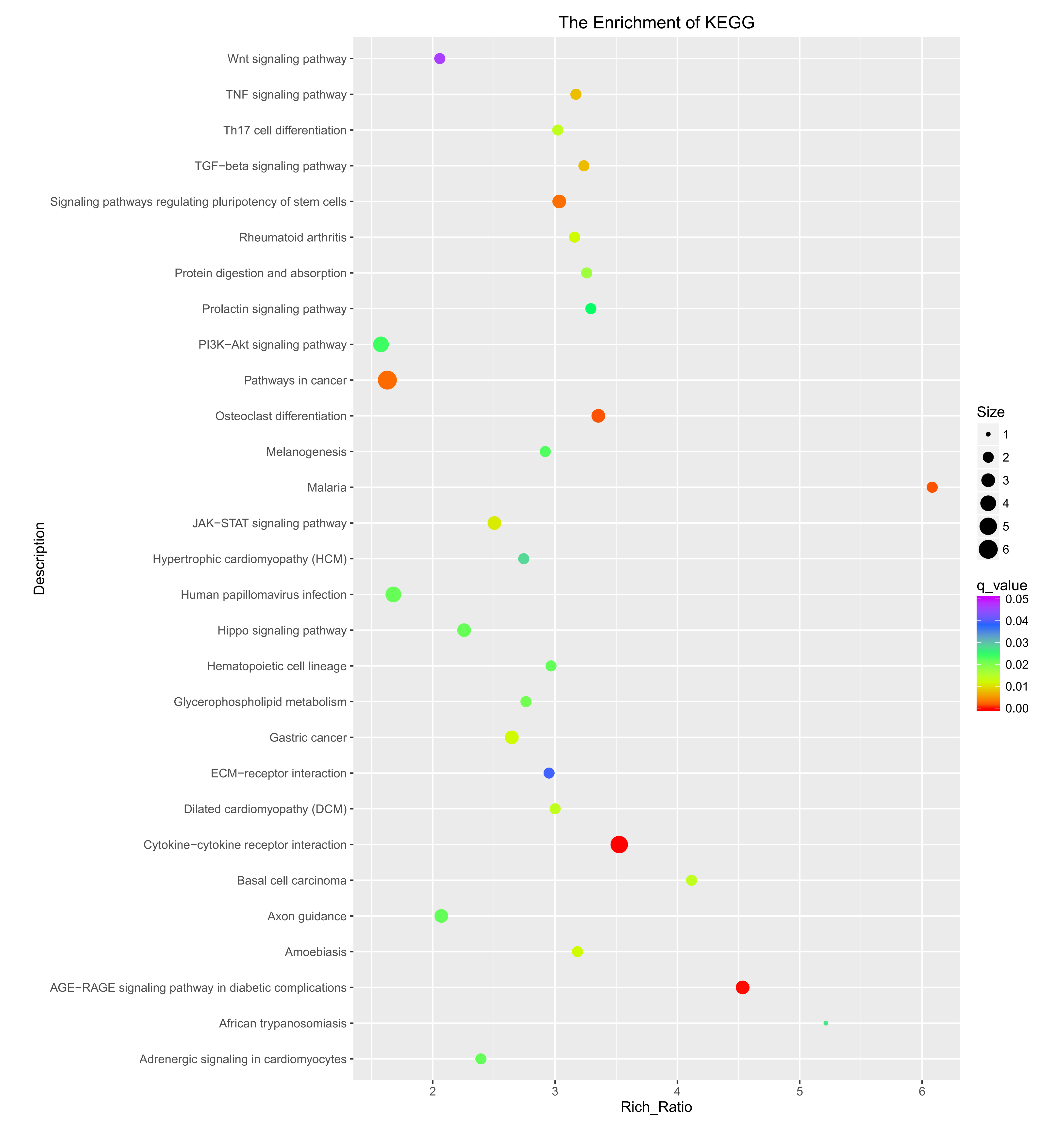

Supplement: Supplementary file 2 [file Image3.JPEG]

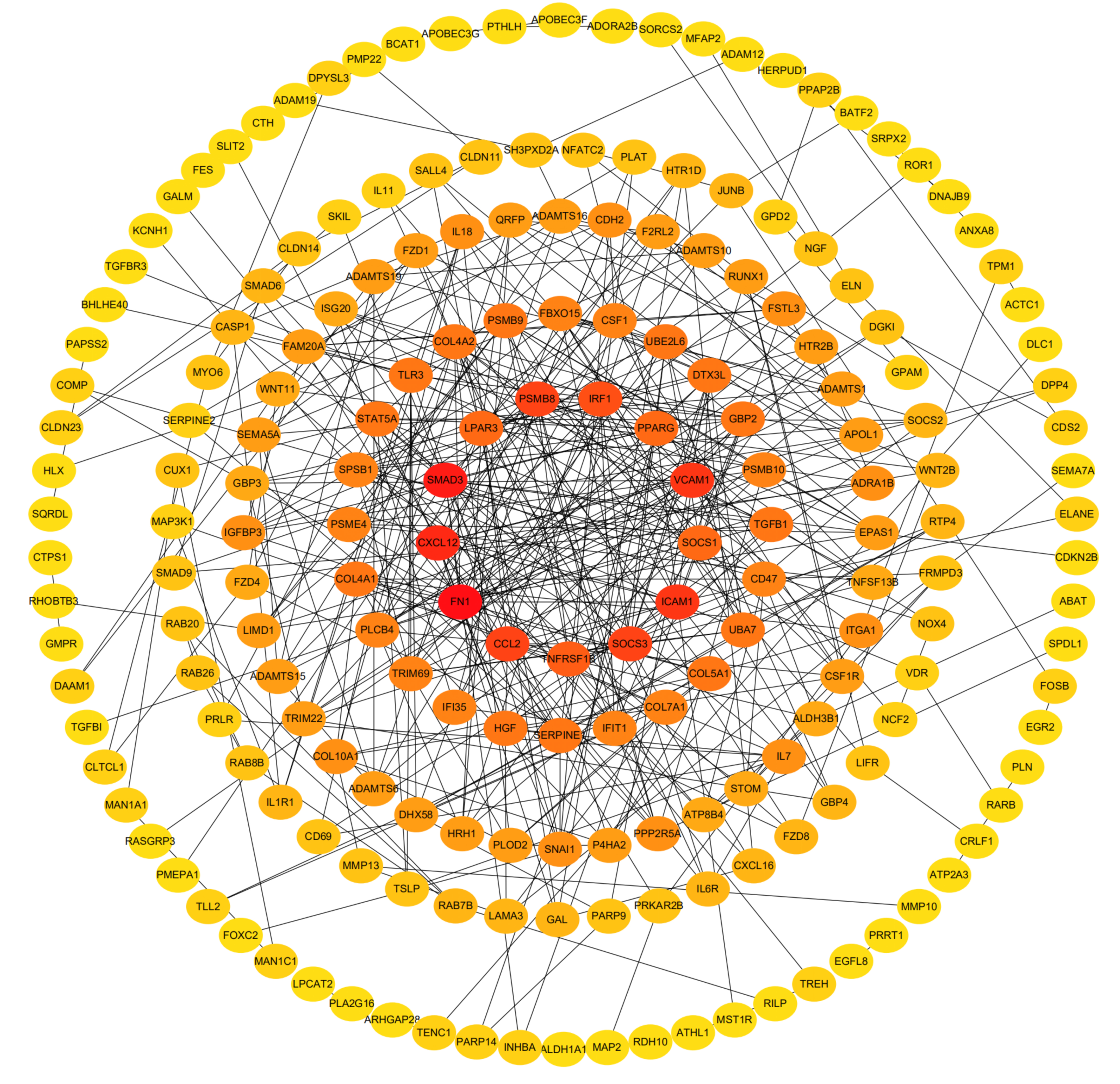

Supplement: Supplementary file 4 [file Image4.JPEG]

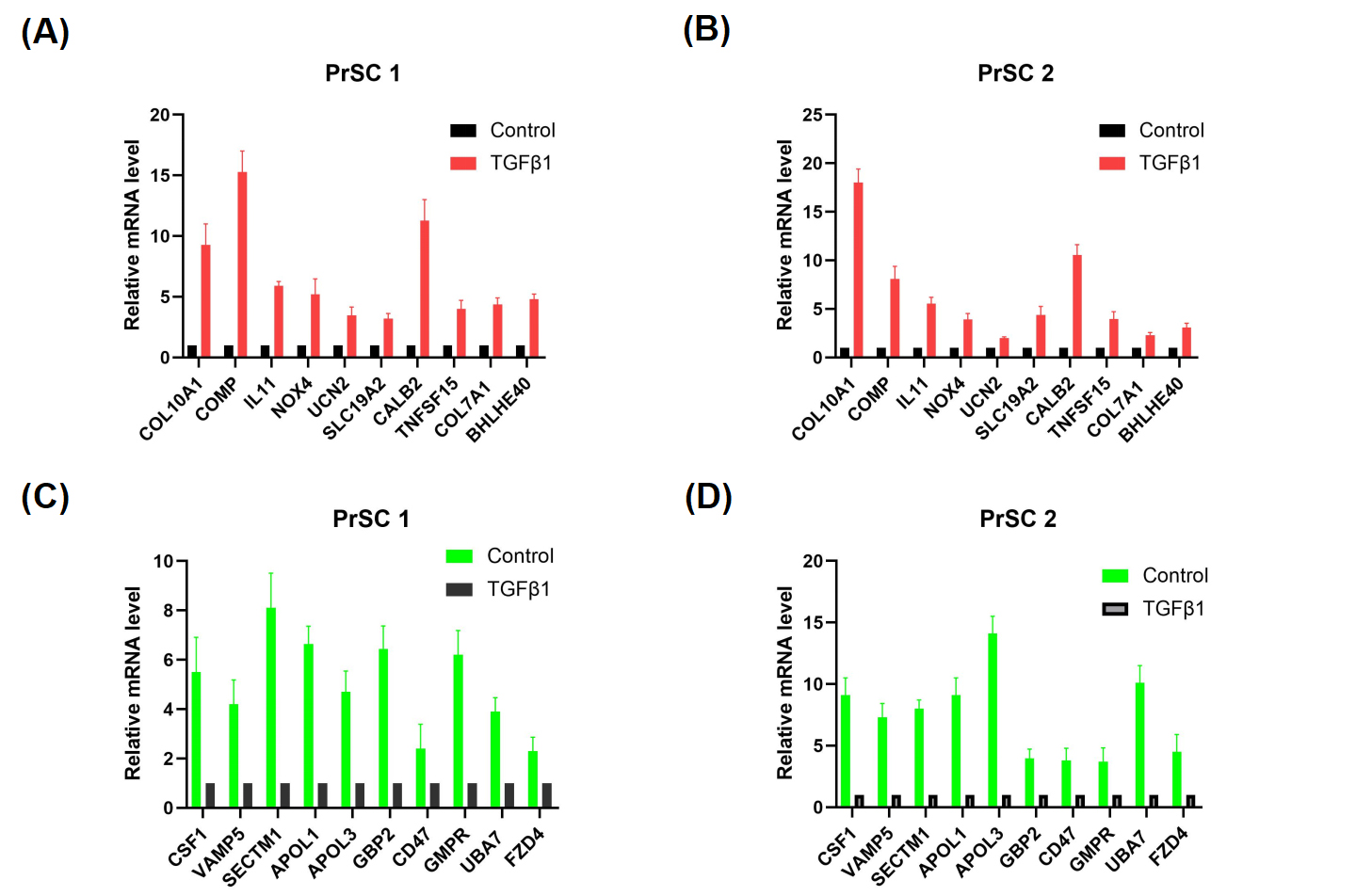

Supplement: Supplementary file 5 [file Image2.JPEG]

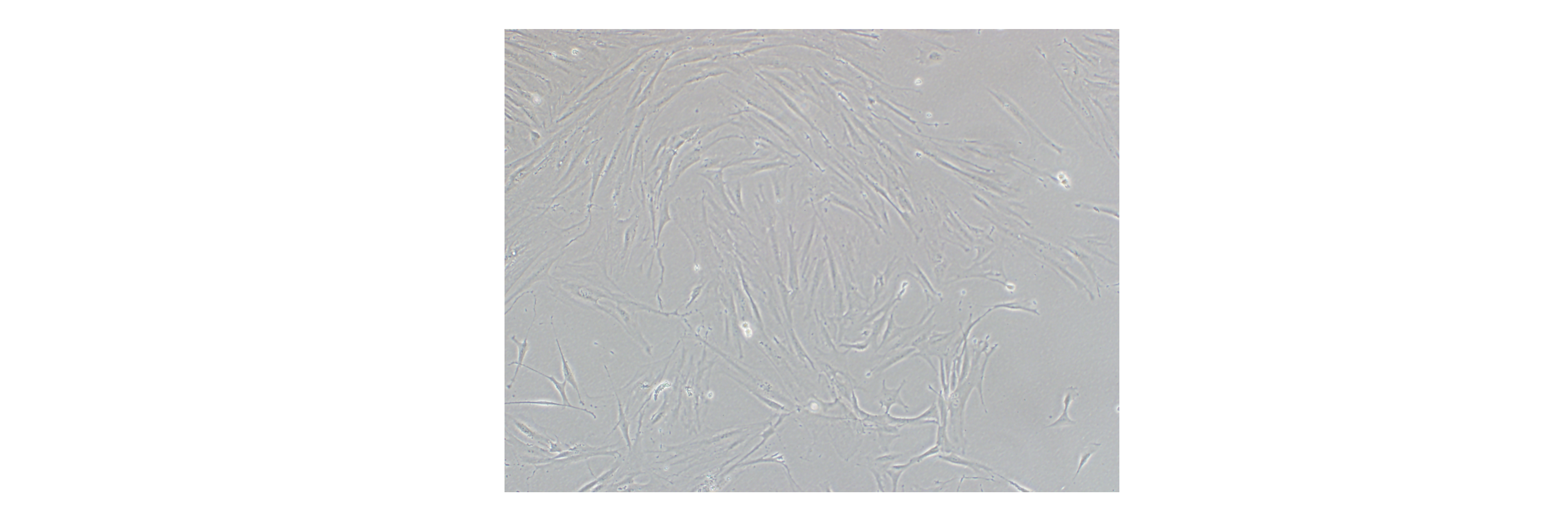

Supplement: Supplementary file 6 [file Image1.TIF]
