# Supplementary material for: RNA sequencing and integrative analysis reveal pathways and hub genes associated with TGFβ1 stimulation on prostatic stromal cells
Source: Front Genet. 2022 Aug 12;13:919103. doi: 10.3389/fgene.2022.919103 (PMC9412917; doi:10.3389/fgene.2022.919103)
Supplement: Supplementary file 7 [file Table3.DOCX]

**Supplementary Table 3** – The hub genes in the protein–protein interaction network (node degree ≥10)

| Gene symbol | Degree |
| --- | --- |
| FN1 | 23 |
| SMAD3 | 16 |
| CXCL12 | 15 |
| VCAM1 | 14 |
| ICAM1 | 14 |
| PSMB8 | 13 |
| SOCS3 | 13 |
| CCL2 | 13 |
| IRF1 | 12 |
| TNFRSF1B | 11 |
| SOCS1 | 10 |
| PPARG | 10 |
| LPAR3 | 10 |
